# A Multi-Omics Approach to Evaluate the Quality of Milk Whey Used in Ricotta Cheese Production

**DOI:** 10.3389/fmicb.2016.01272

**Published:** 2016-08-17

**Authors:** Eleonora Sattin, Nadia A. Andreani, Lisa Carraro, Rosaria Lucchini, Luca Fasolato, Andrea Telatin, Stefania Balzan, Enrico Novelli, Barbara Simionati, Barbara Cardazzo

**Affiliations:** ^1^BMR GenomicsPadova, Italy; ^2^Department of Comparative Biomedicine and Food Science, University of PadovaPadova, Italy; ^3^Istituto Zooprofilattico Sperimentale delle VeneziePadova, Italy

**Keywords:** milk whey, microbial community, volatile compounds, spoilage, food safety

## Abstract

In the past, milk whey was only a by-product of cheese production, but currently, it has a high commercial value for use in the food industries. However, the regulation of whey management (i.e., storage and hygienic properties) has not been updated, and as a consequence, its microbiological quality is very challenging for food safety. The Next Generation Sequencing (NGS) technique was applied to several whey samples used for Ricotta production to evaluate the microbial community composition in depth using both RNA and DNA as templates for NGS library construction. Whey samples demonstrating a high microbial and aerobic spore load contained mostly *Firmicutes*; although variable, some samples contained a relevant amount of *Gammaproteobacteria*. Several lots of whey acquired as raw material for Ricotta production presented defective organoleptic properties. To define the volatile compounds in normal and defective whey samples, a headspace gas chromatography/mass spectrometry (GC/MS) analysis was conducted. The statistical analysis demonstrated that different microbial communities resulted from DNA or cDNA library sequencing, and distinguishable microbiota composed the communities contained in the organoleptic-defective whey samples.

## Introduction

Whey contains valuable components. Therefore, whey has currently several commercial uses. Until some years ago, whey was used only for Ricotta cheese production and as animal feed. To date, a large number of whey-based beverages have been presented to the markets (Özer and Kirmaci, 2010; Pescuma et al., 2010). Moreover, whey proteins are widely used ingredients in the food industry due to their excellent nutritive and functional properties. Whey protein isolates are often used as a nutritional supplement. Additionally, their ability to form gels capable of holding water, lipids, and other components while providing textural properties makes them indispensable in the formulation of many foods, such as processed meat, dairy and bakery products (Kinsella and Whitehead, 1989; de Wit, 1998; Outinen et al., 2010). The exact composition of whey depends by the cheesemaking process, but the common components are proteins, peptides, lactose, vitamins, minerals and fat traces (de Wit, 1998; Outinen et al., 2010). The use of different starter cultures in the cheesemaking process to produce a variety of cheeses results in liquid whey with different mineral contents, protein concentrations, and lactose contents, providing different functional and flavor properties. Off flavors present in whey products may carry through into ingredient applications and negatively affect consumer acceptance (Liaw et al., 2011).

Due to its high nutritional content, milk can support a diverse and complex microbiota, a large part of which can be directly transmitted to whey during cheesemaking. These microorganisms enter milk from a variety of sources and once in milk play a number of roles, such as facilitating dairy fermentation (e.g., *Lactococcus, Lactobacillus, Streptococcus, Propionibacterium*), causing spoilage (e.g., *Pseudomonas, Clostridium, Bacillus* and other spore-forming or thermoduric microorganisms), promoting health (e.g., *Lactobacilli* and *Bifidobacteria*) or causing disease (e.g., *Listeria, Salmonella, Escherichia coli*, and *Campylobacter*) (Quigley et al., 2011, 2013; Vacheyrou et al., 2011). Most of these bacteria can survive the cheesemaking process, and some survive the eventual subsequent pasteurization of the whey. However, despite its high nutritional and economic value, whey is considered to be a by-product of cheese production. Therefore, the regulation of whey management (i.e., storage and hygienic properties) has not been updated, although it is used as a raw material in several food chains. The microbiological quality of the raw material is a very challenging aspect for the production of safe food with an extended shelf life (Lo et al., 2016).

The off flavor due to the starter cultures used for cheesemaking can be increased by improper storage and thermal abuse. The starter bacteria (in particular *Lactococcus lactis*) and the psychrotrophic microflora commonly present in milk (*Pseudomonas* spp. and aerobic spore formers) can grow in pasteurized whey that is not adequately stored and produce modifications in the biochemical composition of whey and defects in flavor through fermentation or oxidation (Campbell et al., 2011). These defects and modifications in the raw material could have a relevant effect on the taste, quality and safety of the final product.

Most of our knowledge with respect to the identity of the microorganisms that are present in raw milk and in the resultant whey and dairy products has been gained through phenotypic methods (Quigley et al., 2011). These testing methods are the standard industrial quality analysis but they are usually labor-intensive, time-consuming and frequently have insufficient discriminatory power. More recently, several more rapid, high-throughput tests based on DNA or RNA analysis that mostly rely on the application of polymerase chain reaction (PCR) technology have been used to confirm the results generated through traditional tests, and their ability to serve as alternatives to culture-based analysis is increasingly being appreciated. One of the key benefits of replacing the culturing step relates to the fact that many microorganisms are difficult to isolate using common culturing methods, potentially leading to a significant underestimation of the microbial communities (Quigley et al., 2013).

NGS platforms have made it possible to recover DNA or cDNA sequence data directly from environmental samples, thereby avoiding the culture step. 16S rRNA sequences are clustered into similarity groups defined as Operational Taxonomic Units (OTUs) and classified with high confidence through comparisons against the 16S rRNA sequence database to provide a more complete description of microbial communities, microbial community interactions and evolution (Loman et al., 2012; Shokralla et al., 2012). New platforms have recently been made available; as a result, this culture-independent method is producing a larger volume of data at a price that is substantially decreasing, which may allow its application in food microbiology to become a tool for diagnostic routine testing in the near future. These aspects open the possibility of increasing the sample size and may allow the analysis of technical or biological replicates for each sample to increase the ability to predict the development of microbial populations in foods (Metzker, 2010).

In the present study, a multi-omics approach (NGS on DNA and RNA, volatile compounds analysis) was applied to evaluate the microbial and chemical qualities of several lots of whey used for Ricotta production. Some of these lots presented defective organoleptic properties. The NGS approach was applied to provide a very comprehensive view of the microbial population composition of whey by comparing normal and defective lots. The application of the same methods to both DNA and cDNA templates allowed us to describe the living microbiota in whey. The microbial community composition and headspace compound analysis were integrated to provide a more complete picture of the quality of the whey samples.

## Materials and methods

### Samples

The sampling included eight lots of fresh pasteurized whey (PW) and nine lots of frozen pasteurized whey (PWF). All of the samples listed in Table 1 were collected from the Ricotta factory “Elda” (Vestenanova, VR, Italy) and originated from several cheese factories located in northeastern Italy and Austria. In the Ricotta factory, the whey samples have an expiry time of 4 days at 10°C (all samples were collected on day 3).

**Table 1:** **Samples, analysis and microbiological counts**.

**Sample**	**Lot/date**	**Analysis**	**TMC (CFU/ml)**	**Bacilli (CFU/ml)**	**Aerobic spores (CFU/ml)**	**Pseudomonas (CFU/ml)**
Pasteurized whey	1PW (10/2013)	M	1.25E+06	3.6E+04	1.02E+03	nd
	2PW (10/2013)	M	9.80E+06	1.35E+06	2.72E+02	nd
	3PW (12/2013)	MS	7.80E+07	4.20E+07	1.20E+03	nd
	4PW (12/2013)	MS	2.10E+06	3.00E+06	3.29E+04	nd
	5PW (02/2014)	M+NGS (RNA)	4.81E+06	nd	6.36E+03	5.21E+04
	6PW (02/2014)	M+NGS (RNA)	8.36E+06	nd	1.85E+04	2.11E+07
	7PW (07/2014)	M+NGS (RNA)	3.20E+07	nd	4.84E+04	5.45E+06
	8PW (07/2014)	M+NGS (RNA)	1.31E+08	nd	4.18E+05	8.73E+07
Frozen pasteurized whey (Summer 2013)	1PWF	NGS (DNA)+GC	–	–	–	–
	2PWF	NGS (DNA)	–	–	–	–
	3PWF	NGS (DNA)+GC	–	–	–	–
	4PWF	NGS (DNA)+GC	–	–	–	–
	5PWFnc	NGS (DNA)+GC	–	–	–	–
	6PWFnc	NGS (DNA)+GC	–	–	–	–
	7PWFnc	NGS (DNA)+GC	–	–	–	–
	8PWFnc	NGS (DNA)	–	–	–	–
	9PWFnc	NGS (DNA)	–	–	–	–

*M, Microbiological counts; MS, Microbiological counts +Sanger sequencing on isolated colonies; NGS, Next Generation Sequencing (rRNA 16S gene library); GC, Gas chromatography; nd, not determinate*.

The eight lots of fresh pasteurized whey (PW) were used in Ricotta production. All of the Ricotta lots were sampled and analyzed for the evolution of microbial communities throughout their shelf-life (Sattin et al., 2016). The frozen pasteurized whey (PWF) samples were collected by the factory during the summer of 2013 and classified as normal or defective based on their organoleptic properties. The organoleptic evaluations were performed based on the internal standard procedures in the factory. The defective lots resulted in a grass/cooked defect in smell and taste, but the microbiology counts and pH were reported as normal by the factory (data not shown). The normal lots were used for Ricotta production, and the defective lots were excluded from production. The factory stocked frozen whey lots from the summer of 2013. Nine PWF lots were sampled, including four normal (PWF) and five defective lots (hereafter called PWFnc to indicate non-compliant samples).

Table 1 presents the collected whey lots and the analysis conducted on each lot.

### Culture-dependent methods

The collected samples were transported to the laboratory in refrigerated containers (4°C). Then, 20 mL of PW were aseptically added to 180 mL of buffered peptone water (Biokar Diagnostics, Pantin Cedex, France) and serially diluted in the same solution. Samples were analyzed for aerobic mesophilic microorganisms (TMC) on Plate Count Agar containing skimmed milk (milk*PCA*, Biokar Diagnostics). Additionally, the vegetative forms of bacilli were assessed on Mannitol Egg Yolk Polymyxin Agar (MYP agar; Sacco, Como, Italy) and *Pseudomonas* was assessed on CFC *Pseudomonas* Agar Base (CFC PAB; Oxoid Basingstoke, UK). The plates were incubated at 30°C for 24–48 h except for CFC/PAB, which was incubated at 22°C for 24–48 h. For the sulfite reductor *Clostridium*, the samples were plated on sulfite polymixin sulfadizine agar (SPS, Sacco) and incubated at 37°C for 24–48 h in under anaerobic conditions. For aerobic spore enumeration, 10 mL of 1:10 diluted samples were incubated for 10 min at 80°C, plated in Plate Count Agar supplemented with 0.2% starch (PCA and Starch, Biokar Diagnostics) and incubated at 30°C for 2–5 days. For anaerobic spore enumeration, 10 mL of 1:10 diluted samples were incubated for 10 min at 80°C, plated in sulfite iron agar (SIA, Sacco) and incubated at 37°C for 2–5 days under anaerobic conditions. Between three and five isolates were randomly picked from the MilkPCA, MYP and PCA+starch counting plates (3PW and 4PW) and cultured on the corresponding media. Isolates were identified by combining PCR with 16S–23S rRNA gene spacer analysis (RSA), species-specific primers and 16S rRNA gene sequencing. For DNA extraction, a single colony from a fresh culture was resuspended in 100 μl of nuclease-free water, vortexed at high speed for 5 s, and incubated at 94°C for 10 min. The tube was vortexed again and centrifuged for 2 min at 14,000 rpm. The supernatant was transferred to a fresh tube and stored at −20°C. The concentration and quality of the extracted DNA were determined using a NanoDrop spectrophotometer. Approximately 25 ng of DNA was subjected to RSA analysis with the primers G1 and L1 (Coppola et al., 2001). The RSA amplification profiles were separated into different clusters and compared with reference strains. According to the RSA profile comparisons, two samples for each cluster group and the undefined samples (not amplified or the band resolution were not clear) were submitted for partial 16S rRNA gene amplification with the primers 16SbactF and 16SbactR (Nadkarni et al., 2002). The amplified fragments were sequenced, and the obtained sequences were aligned with the closest sequences available in the GenBank database (98% homology; https://blast.ncbi.nlm.nih.gov/Blast.cgi).

### Culture-independent methods

For DNA extraction, 2 mL of PWF or PWFnc were collected and centrifuged at 13,500 rpm for 10 min to separate the supernatant from the bacterial cells. The pellets were resuspended in 1 mL of PBS (phosphate-buffered saline) and centrifuged at 5000 rpm for 5 min. After discarding the supernatant, 40 μl of proteinase K and 400 μl of Lysis buffer (Invitek, Berlin, Germany) were added to the pellets, and the tubes were incubated at 56°C on a shaker for 2 h. DNA extraction was performed using the Spin Tissue Mini Kit Invisorb (Invitek, Berlin, Germany) following the manufacturer's instructions. The elution step was repeated twice to increase the total yield in a final volume of 100 μl.

For RNA extraction, 5 mL of the 5PW-6PW-7PW-8PW samples were diluted 1:10 in MRD (maximum recovery diluent; 1 g/l peptone and 0.75% NaCl) and centrifuged at 8000 rpm for 5 min. The pellets were resuspended in 700 μl of buffer RTL, 3 μl of β-mercaptoethanol (Sigma-Aldrich, St. Louis, MO, USA) and 250 μl of zirconia beads. The samples were homogenized with a Ribolyzer homogenizer (Hybaid, Thermo Scientific, Waltham, MA, USA) for 20 s at speed 4. The homogenized samples were centrifuged for 10 s at maximum speed; then, 700 μl of the supernatants were collected, and RNA was extracted with the RNeasy Tissue Mini Kit (Qiagen, Hilden, Germany) following the manufacturer's instructions. The reverse transcription step was performed in a final volume of 20 μl with the Superscript II Reverse Transcriptase Kit (Invitrogen, Carlsbad, CA, USA) following the manufacturer's instruction.

For NGS sequencing, the V3–V4 regions of the 16S rRNA gene were amplified using the primers 331F 5′-TCCTACGGG AGGCAGCAGT-3′ and 797R 5′-GGA CTACCAGGGTATCTAATCCTGTT (Nadkarni et al., 2002). The primers were modified with a forward overhang (5′-TCGT CGGCAGCGTCAGATGTGTATAAGAGA CAG−[locus−specific sequence]-3′) and a reverse overhang (5′- GTCTCGTGGGCTCGGAGATGTGTATAAGAG ACAG−[locus−specific sequence]-3′), which was necessary for dual index library preparation. The library was run on the Illumina MiSeq (San Diego, California, USA) using the 2 × 300 bp paired-end approach.

The sequencing reads were filtered for average quality (*Q* > 30), and R1 and R2 were merged using FLASH with default parameters (Magoč and Salzberg, 2011). Qiime version 1.8 was used to perform the full analysis of OTUs selected for the statistical analysis using the *pick_closed_reference_otus* wrapper from the Greengenes reference database (13.8 version, Caporaso et al., 2010). The following wrappers were exploited for the other steps: *assign_taxonomy* (to assign Greengenes taxonomy to OTUs), *make_otu_table* (to create a biom file with OTUs and taxonomy)*, compute_core_microbiome* (to calculate the core microbiome), *summarize_taxa_through_plots* (to produce the taxonomic files and charts), *alpha_rarefaction* and *beta_diversity_through_plots* (to assess the alpha- and beta-rarefaction diversity indices, respectively), and *group_significance.py* (to identify the differentially expressed OTUs in the sample groups based on the non-parametric Kruskal-Wallis test). The sequence data file was deposited in the SRA database with accession number SRP070771.

### SPME-GC/MS analysis of volatile compounds and the correlation with the microbial community composition

Whey samples (10 mL) were placed into 22 mL glass vials (20 × 72 mm, d × h) sealed with an aluminum cap. Solid-phase microextraction (SPME) was performed using a 100 μm polydimethylsiloxane (PDMS) fiber (Supelco, Bellefonte, PA, USA). The samples were placed under stirring and brought to 40°C in 5 min; then, the PDMS fibers were exposed to the vial's headspace for 30 min.

GC/MS analysis of the volatile compounds adsorbed onto the SPME fiber was performed using a Shimadzu GC2010 gas chromatograph equipped with a QP2010 mass selective detector (Shimadzu Italia, Milano, Italy). A ZB5 column (5% phenyl-, 95% dimethylpolysiloxane, 60 m, 0.25-mm internal diameter, 0.25-μm film thickness; Phenomenex Inc., Torrance, CA, USA) and helium flowing at constant speed of 35 cm min^−1^ were used as the capillary column and carrier gas, respectively. The injector was operated in the on-column mode at 310°C. The SPME fiber was retained in the injector for 30 s for thermal desorption. Initially, the column was maintained at 35°C for 4 min, heated to 300°C at a rate of 5°C min^−1^ and held at the final temperature for 10 min. The mass spectrometric (MS) conditions were as follows: source at 220°C; acquisition in the electron-impact (EI) mode (70 eV) with 2 scans s^−1^; and mass/charge (m/z) range 29–450. The temperature of the transfer line was held constant at 220°C. Peak identification was performed by comparing the retention times and the mass spectra of the eluted compounds to those in the NIST library (Standard Reference Data, NIST, Gaithersburg, MD, USA). The area of each identified compound was subjected to a normalization process, and its value was transformed into a percentage.

A hierarchical clustering analysis of the composition of the headspace volatile compounds of the whey samples was performed in the R environment (http://www.rstudio.com). The dissimilarity matrix was created using the Euclidean distance. The most interesting bacteria from the point of view of the production process and food quality issues were chosen for the subsequent statistical analysis. Relative abundance of these bacteria was obtained from the output file of summarize_taxa wrapper of Qiime pipeline and merged with the abundance table of volatile compounds. The {cor} function in R studio with non-parametric Spearman's rho statistic option was used to estimate the correlation between chemical compounds and bacterial taxon abundances. These measurements were plotted in a heatmap representation using the dplyr, tdyr, and ggplot2 R packages.

## Results

### Preliminary analysis of whey using culture-dependent methods

The whey samples were analyzed using culture-dependent methods to describe the general microbiological condition (TMCs, spore-forming bacteria and spoilers such as *Pseudomonas*).

The TMCs for both lots was comprised between 10^6^ and 10^8^ CFU/mL, and the aerobic spores numbered between 10^2^ and 10^5^ CFU/mL, suggesting that seasonality did not influence the bacterial abundance. No vegetative cell or spore growth was detected in the SPS and SIA broths. All of the counts are reported in Table 1. The 1PW and 2PW lot analysis evaluated the counts without any additional identification of the colonies, whereas a total of 200 colonies were selected from the 3PW and 4PW lot plates for RSA screening. The identification of the 29 isolates reported in Table S1 demonstrated a moderate degree of biodiversity in the whey microflora.

### Culture-independent methods used to evaluate the whey microflora biodiversity

Microbial community profiles of the whey samples were assessed by 16S rRNA gene sequencing using the Illumina technology. DNA was extracted from the PWF or PWFnc and used as a template for library construction. In addition to describing the living microbiota in the whey, two lots were sampled in winter and two in summer (PW). RNA was extracted, reverse transcribed and used for library construction. The MiSeq runs produced 5213557 extended fragments after the quality filtering and FLASH steps with an average length of 466 bp (±2.2184 bp), including the V3 and V4 regions of the 16S rRNA gene. A total of 1154 OTUs with 97% similarity were obtained after OTU picking. The diversity indices and coverage are presented in Table 2. The highest tree variability (phylogenetic diversity, PD, and whole tree value) was observed in the summer PW (7PW and 8PW). High values were found in all PW samples (range = 11.01–25.41) compared to the PWF samples (range = 9.04–16.78), indicating that the active communities sampled using RNA as the template presented major biodiversity. In accordance with this result, the species richness estimated by the Chao1 index (Chao and Bunge, 2002) was higher for the PW samples (range = 326.09–871.37) than for the PWF samples (range = 189.29–430.98). Good's coverage, which is an estimator of the sampling completeness, calculates the probability that a randomly selected amplicon sequence from a sample has been already sequenced and indicated a level of sequencing that was adequate to identify the majority of the diversity in the dairy samples.

**Table 2:** **Diversity indices, coverage and number of reads of whey sample sequencing**.

**Sample**	**PD whole tree**	**Chao1**	**Good's coverage (%)**	**Reads number**
5PW	17.72	517.89	99.81	65197
6PW	11.01	326.09	99.92	79700
7PW	21.29	713.20	99.96	411094
8PW	25.41	871.37	99.97	481639
1PWF	12.21	315.50	99.99	267278
2PWF	9.84	242.53	99.98	151600
3PWF	9.04	189.29	100.00	600077
4PWF	12.99	348.28	99.99	338207
5PWFnc	15.13	390.88	99.99	335405
6PWFnc	10.52	236.54	99.99	339146
7PWFnc	13.56	364.79	99.99	775811
8PWFnc	16.78	430.98	99.99	509753
9PWFnc	10.45	254.54	99.98	132974

**Figure 1:**
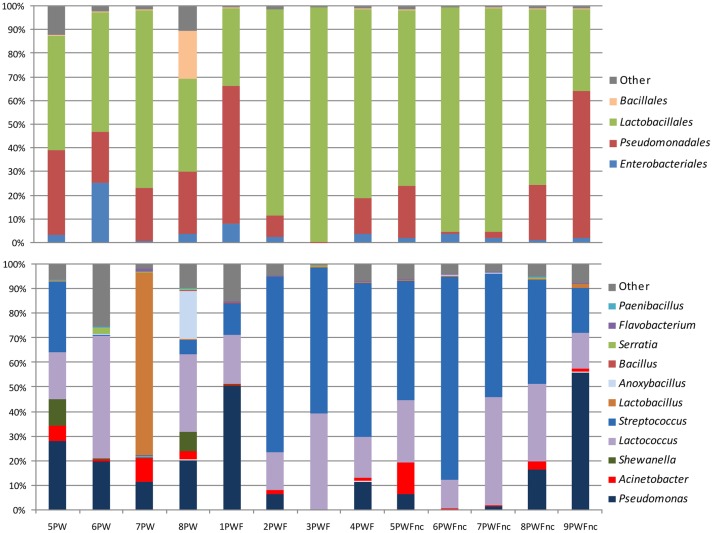
**Distribution of orders and genera in the whey microbial community samples**.

The most common phylum was *Firmicutes* (range = 33.09–99.57%), and it was represented by the orders *Lactobacillales* and *Bacillales*, which were a consistent part of the community in all samples as highlighted in the top bars in Figure 1. The presence of components of *Proteobacteria* was more variable among the samples (range = 0.41–66.82%). Most of the *Proteobacteria* belonged to the *Gammaproteobacteria* class, *Pseudomonadales* order. The phyla *Bacterioidetes* (range = 0.02–1.09%) and *Actinobacteria* (range = 0.00–0.08%) represented the minority components of all communities. Only 53 OTUs were part of the core microbiome (shared between 100% of the samples); these OTUs were comprised of the *Streptococcaceae* (11 OTUs), *Pseudomonadaceae* (8 OTUs), *Moraxellaceae* (17 OTUs), *Enterobacteriaceae* (14 OTUs), *Lactobacillaceae* (1 OTU), *Aeromonadaceae* (1 OTU), and *Weeksellaceae* (1 OTU) families. Generally, the most abundant bacterial genera (shown in the bottom bars in Figure 1) were *Streptococcus* (range = 0.13–82.62%), *Lactococcus* (range = 0.65–49.85%)*, Pseudomonas* (range = 0.02–56.16%), *Acinetobacter* (range = 0.18–12.92%), and *Shewanella* (range = 0.00–10.92%). *Lactobacillus* and *Anoxybacillus* were found in only a few samples at high percentages (74.46% in 7PW and 19.90% in 8PW, respectively). Many other genera, such as *Bacillus, Serratia, Flavobacterium*, and *Paenibacillus*, were present with a less than 1% abundance.

**Figure 2:**
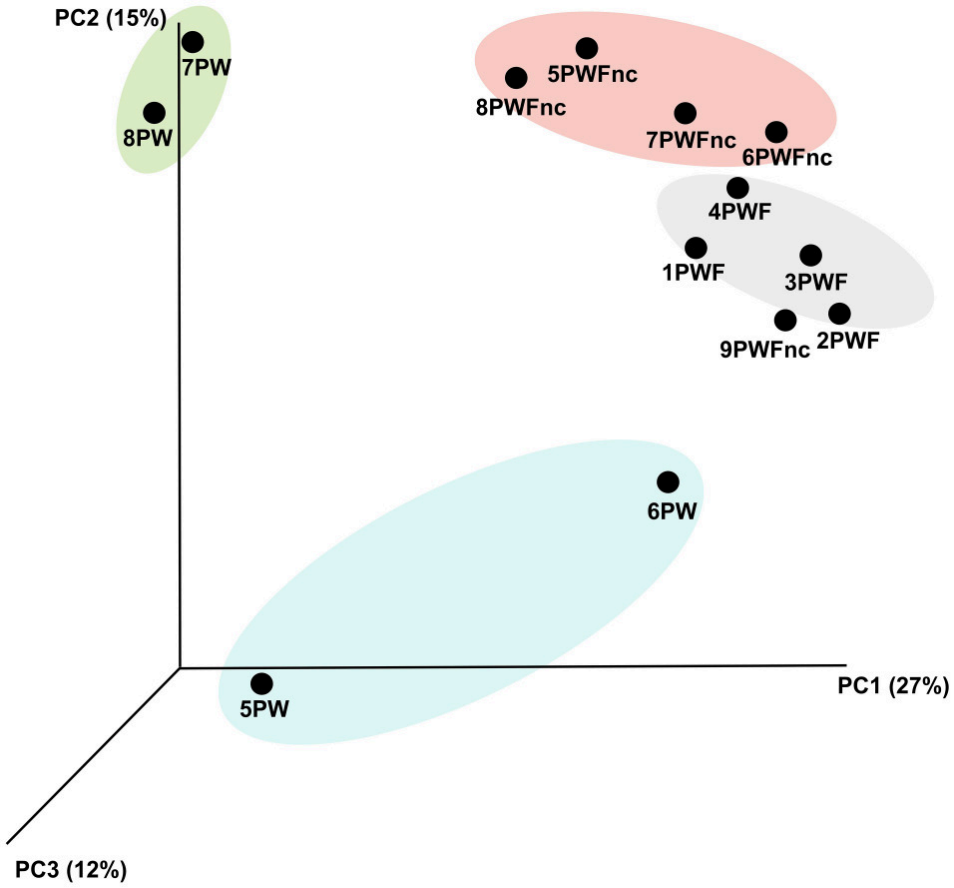
**The 3D unweighted PCoA analysis demonstrated the b-diversity among the whey samples**. Different colors indicate different groups of samples. Yellow, summer RNA samples. Light blue, winter RNA samples. Pink, defective DNA samples. Green, normal DNA samples.

The definition of the β-diversity was performed using a 3D unweighted Principal Coordinates Analysis (PCoA) to highlight the differences in sample compositions (presented in Figure 2). The principal coordinates 1, 2, and 3 explained 27, 15, and 12% of the variation, respectively. Four main clusters were observed as follows: the summer PW (7PW–8PW), winter PW (5PW and 6PW), PWF, and PWFnc samples, although 9PWFnc was located among the PWF samples.

The β-diversity among the whey samples was also evaluated using a Kruskall-Wallis analysis. The differences in the microbial composition were investigated by comparing samples belonging to two groups (1 and 2) on the basis of three parameters: *template* (1. DNA and 2. RNA), *season* (1. summer and 2. winter) and *organoleptic properties* (1. normal and 2. defective). First, the analysis was performed considering all 13 samples together. Then, to evaluate the effect of each single parameter, we analyzed subgroups of samples as follows: *template* considering only summer samples, *season* considering only RNA samples and *organoleptic properties* considering only DNA samples. The analysis is presented in Table 3, and the complete list of the OTUs that exhibited different presences/quantities in the groups of samples is presented in Tables 4, 5. The statistical analysis demonstrated a significant difference for *template* when only the summer samples were considered (*p* < 0.01). The RNA/DNA communities presented 37 overrepresented OTUs in the RNA community, most of which were completely absent in the DNA community (32/37). Twenty of the 37 total OTUs were identified as *Gammaproteobacteria* taxa, of which 8 OTUs were *Pseudomonas* (see Table 4). A significant difference was also observed for *organoleptic properties* when all samples or only the DNA community samples were considered (*p* < 0.05). Most of the OTUs reported were overrepresented in the PWFnc samples (15/18 or 10/14, Table 5). In the comparison using only the DNA samples that eliminated the effect of the DNA/RNA template, 8/14 OTUs overrepresented in defective whey were identified as *Gammaproteobacteria* (4/8 were genus *Acinetobacter*).

**Table 3:** **Parameters and groups (described as category, number of samples included in the category and sample names) considered in the Kruskall-Wallis analysis of β-diversity among whey microbial community composition**.

**Parameters**	**Group 1**			**Group 2**			**Results**	
	**Categories**	**n**	**Samples**	**Categories**	**n**	**Samples**	***p***	**OTU n**
Template	DNA	9	All PWF+PWFnc	RNA	4	All PW	NS	–
Template (only Summer)	DNA	9	All PWF+PWFnc	RNA	2	7PW+8PW	<0.01	37
Season	Summer	11	7PW+8PW+ All PWFnc	Winter	2	5PW+6PW	NS	–
Season (only RNA)	Summer	2	7PW+8PW	Winter	2	5PW+6PW	NS	–
Organoleptic properties	Normal	8	All PWF+PW	Defective (nc)	5	All PWFnc	<0.05	18
Organoleptic properties (only DNA)	Normal	4	All PWF	Defective (nc)	5	All PWFnc	<0.05	14

*The results are reported as probability of difference among communities and number of differently represented OTUs. NS, Not Significant*.

**Table 4:** **List of OTUs resulted differently represented in RNA and DNA communities**.

**OTU**	**RNA_mean**	**DNA_mean**
**TEMPLATE (SUMMER SAMPLES) *p* < 0.01**		
p__Actinobacteria; c__Actinobacteria; o__Actinomycetales; f__Micrococcaceae	1	0
p__Actinobacteria; c__Actinobacteria; o__Actinomycetales; f__Propionibacteriaceae; g__Propionibacterium; s__acnes	7.5	0
p__Bacteroidetes; c__Flavobacteriia; o__Flavobacteriales; f__[Weeksellaceae]; g__Chryseobacterium	4.5	0
p__Bacteroidetes; c__Flavobacteriia; o__Flavobacteriales; f__[Weeksellaceae]; g__Cloacibacterium	24	0
p__Bacteroidetes; c__Flavobacteriia; o__Flavobacteriales; f__Flavobacteriaceae; g__Flavobacterium	11	0
p__Bacteroidetes; c__Sphingobacteriia; o__Sphingobacteriales; f__Sphingobacteriaceae; g__Pedobacter	1.5	0
p__Firmicutes; c__Bacilli; o__Lactobacillales; f__Carnobacteriaceae; g__Carnobacterium	6	0.11111111
p__Firmicutes; c__Bacilli; o__Bacillales; f__Staphylococcaceae; g__Staphylococcus; s__epidermidis	12	0
p__Proteobacteria	4.5	0
p__Proteobacteria; c__Alphaproteobacteria; o__Caulobacterales; f__Caulobacteraceae	14	0
p__Proteobacteria; c__Alphaproteobacteria; o__Rhizobiales; f__Bradyrhizobiaceae	9	0
p__Proteobacteria; c__Alphaproteobacteria; o__Rhizobiales; f__Rhizobiaceae; g__Agrobacterium	1.5	0
p__Proteobacteria; c__Alphaproteobacteria; o__Sphingomonadales; f__Sphingomonadaceae; g__Sphingomonas	1	0
p__Proteobacteria; c__Betaproteobacteria; o__Burkholderiales; f__Comamonadaceae	3	0
p__Proteobacteria; c__Betaproteobacteria; o__Burkholderiales; f__Comamonadaceae; g__Delftia	8	0
p__Proteobacteria; c__Gammaproteobacteria; o__Aeromonadales; f__Aeromonadaceae	13.5	0
p__Proteobacteria; c__Gammaproteobacteria; o__Aeromonadales; f__Aeromonadaceae	5.5	0
p__Proteobacteria; c__Gammaproteobacteria; o__Aeromonadales; f__Aeromonadaceae	6	0.11111111
p__Proteobacteria; c__Gammaproteobacteria; o__Aeromonadales; f__Aeromonadaceae	7.5	0
p__Proteobacteria; c__Gammaproteobacteria; o__Aeromonadales; f__Aeromonadaceae	28	0.11111111
p__Proteobacteria; c__Gammaproteobacteria; o__Alteromonadales; f__Shewanellaceae; g__Shewanella	16529.5	0
p__Proteobacteria; c__Gammaproteobacteria; o__Enterobacteriales; f__Enterobacteriaceae	15	0
p__Proteobacteria; c__Gammaproteobacteria; o__Enterobacteriales; f__Enterobacteriaceae	118	0
p__Proteobacteria; c__Gammaproteobacteria; o__Enterobacteriales; f__Enterobacteriaceae	1	0
p__Proteobacteria; c__Gammaproteobacteria; o__Pseudomonadales; f__Moraxellaceae; g__Acinetobacter	1	0
p__Proteobacteria; c__Gammaproteobacteria; o__Pseudomonadales; f__Pseudomonadaceae; g__Pseudomonas	1	0
p__Proteobacteria; c__Gammaproteobacteria; o__Pseudomonadales; f__Pseudomonadaceae; g__Pseudomonas	1	0
p__Proteobacteria; c__Gammaproteobacteria; o__Pseudomonadales; f__Pseudomonadaceae; g__Pseudomonas	10.5	0
p__Proteobacteria; c__Gammaproteobacteria; o__Pseudomonadales; f__Pseudomonadaceae; g__Pseudomonas	16.5	0
p__Proteobacteria; c__Gammaproteobacteria; o__Pseudomonadales; f__Pseudomonadaceae; g__Pseudomonas	15.5	0.22222222
p__Proteobacteria; c__Gammaproteobacteria; o__Pseudomonadales; f__Pseudomonadaceae; g__Pseudomonas	2.5	0.11111111
p__Proteobacteria; c__Gammaproteobacteria; o__Pseudomonadales; f__Pseudomonadaceae; g__Pseudomonas	1	0
p__Proteobacteria; c__Gammaproteobacteria; o__Pseudomonadales; f__Pseudomonadaceae; g__Pseudomonas; s__stutzeri	1	0
p__Proteobacteria; c__Gammaproteobacteria; o__Xanthomonadales; f__Xanthomonadaceae; g__Stenotrophomonas; s__acidaminiphila	3.5	0
p__Proteobacteria; c__Gammaproteobacteria; o__Xanthomonadales; f__Xanthomonadaceae; g__Stenotrophomonas; s__rhizophila	32	0
p__TM7; c__TM7-3	17.5	0
p__TM7; c__TM7-3; o__EW055	2	0

**Table 5:** **List of OTUs resulted differently represented in sample with normal or defective organoleptic properties**.

**OTU**	**PW+PWF_mean**	**PWFnc_mean**
**ORGANOLEPTIC PROPERTIES (ALL SAMPLES) *p* <0.05**		
p__Bacteroidetes; c__Flavobacteriia; o__Flavobacteriales; f__Flavobacteriaceae; g__Flavobacterium; s__succinicans	0.75	10
p__Bacteroidetes; c__Flavobacteriia; o__Flavobacteriales; f__Flavobacteriaceae; g__Myroides	0	4
p__Bacteroidetes; c__Flavobacteriia; o__Flavobacteriales; f__[Weeksellaceae]; g__Chryseobacterium	0.125	24.8
p__Bacteroidetes; c__Flavobacteriia; o__Flavobacteriales; f__[Weeksellaceae]; g__Chryseobacterium	1	28.4
p__Bacteroidetes; c__Flavobacteriia; o__Flavobacteriales; f__[Weeksellaceae]; g__Chryseobacterium	1	13.2
p__Bacteroidetes; c__Flavobacteriia; o__Flavobacteriales; f__[Weeksellaceae]; g__Wautersiella	1.25	26.2
p__Firmicutes; c__Bacilli; o__Lactobacillales; f__Enterococcaceae; g__Enterococcus	1.125	3.4
p__Firmicutes; c__Bacilli; o__Lactobacillales; f__Lactobacillaceae; g__Lactobacillus	0.125	4.4
p__Firmicutes; c__Bacilli; o__Lactobacillales; f__Streptococcaceae; g__Lactococcus	1.75	12.2
p__Firmicutes; c__Bacilli; o__Lactobacillales; f__Streptococcaceae; g__Lactococcus	94.375	107.2
p__Firmicutes; c__Bacilli; o__Lactobacillales; f__Streptococcaceae; g__Lactococcus	1.375	12.4
p__Firmicutes; c__Bacilli; o__Lactobacillales; f__Streptococcaceae; g__Streptococcus	3.25	16.2
p__Proteobacteria; c__Betaproteobacteria; o__Burkholderiales; f__Comamonadaceae; g__Delftia	0	3.6
p__Proteobacteria; c__Gammaproteobacteria; o__Aeromonadales; f__Aeromonadaceae	25.25	22.2
p__Proteobacteria; c__Gammaproteobacteria; o__Enterobacteriales; f__Enterobacteriaceae	3.125	0.2
p__Proteobacteria; c__Gammaproteobacteria; o__Pseudomonadales; f__Moraxellaceae; g__Acinetobacter	0	3
p__Proteobacteria; c__Gammaproteobacteria; o__Pseudomonadales; f__Moraxellaceae; g__Enhydrobacter	26.5	145.6
p__Proteobacteria; c__Gammaproteobacteria; o__Pseudomonadales; f__Pseudomonadaceae; g__Pseudomonas	4.5	0.4
**OTU**	**PWF_mean**	**PWFnc_mean**
**ORGANOLEPTIC PROPERTIES (DNA SAMPLE) *p* <0.05**		
p__Bacteroidetes; c__Flavobacteriia; o__Flavobacteriales; f__[Weeksellaceae]; g__Chryseobacterium	0	28.4
p__Firmicutes; c__Bacilli; o__Lactobacillales; f__Lactobacillaceae; g__Lactobacillus; s__zeae	0	5.2
p__Proteobacteria; c__Betaproteobacteria; o__Burkholderiales; f__Comamonadaceae; g__Comamonas	0	2.6
p__Proteobacteria; c__Gammaproteobacteria; o__Aeromonadales; f__Aeromonadaceae	0.5	22.2
p__Proteobacteria; c__Gammaproteobacteria; o__Aeromonadales; f__Aeromonadaceae; g__Aeromonas	0	3
p__Proteobacteria; c__Gammaproteobacteria; o__Enterobacteriales; f__Enterobacteriaceae	5.25	0.2
p__Proteobacteria; c__Gammaproteobacteria; o__Enterobacteriales; f__Enterobacteriaceae	2.75	0
p__Proteobacteria; c__Gammaproteobacteria; o__Enterobacteriales; f__Enterobacteriaceae	5.25	0
p__Proteobacteria; c__Gammaproteobacteria; o__Pseudomonadales; f__Moraxellaceae	0	2
p__Proteobacteria; c__Gammaproteobacteria; o__Pseudomonadales; f__Moraxellaceae; g__Acinetobacter	0	13.8
p__Proteobacteria; c__Gammaproteobacteria; o__Pseudomonadales; f__Moraxellaceae; g__Acinetobacter	0	10.8
p__Proteobacteria; c__Gammaproteobacteria; o__Pseudomonadales; f__Moraxellaceae; g__Acinetobacter	0.25	9.4
p__Proteobacteria; c__Gammaproteobacteria; o__Pseudomonadales; f__Moraxellaceae; g__Acinetobacter	71.5	674.2
p__Proteobacteria; c__Gammaproteobacteria; o__Pseudomonadales; f__Pseudomonadaceae; g__Pseudomonas	2	0

*In gray are the OTUs overrepresented in normal samples*.

### SPME-GC/MS analysis of the PWF samples

**Figure 3:**
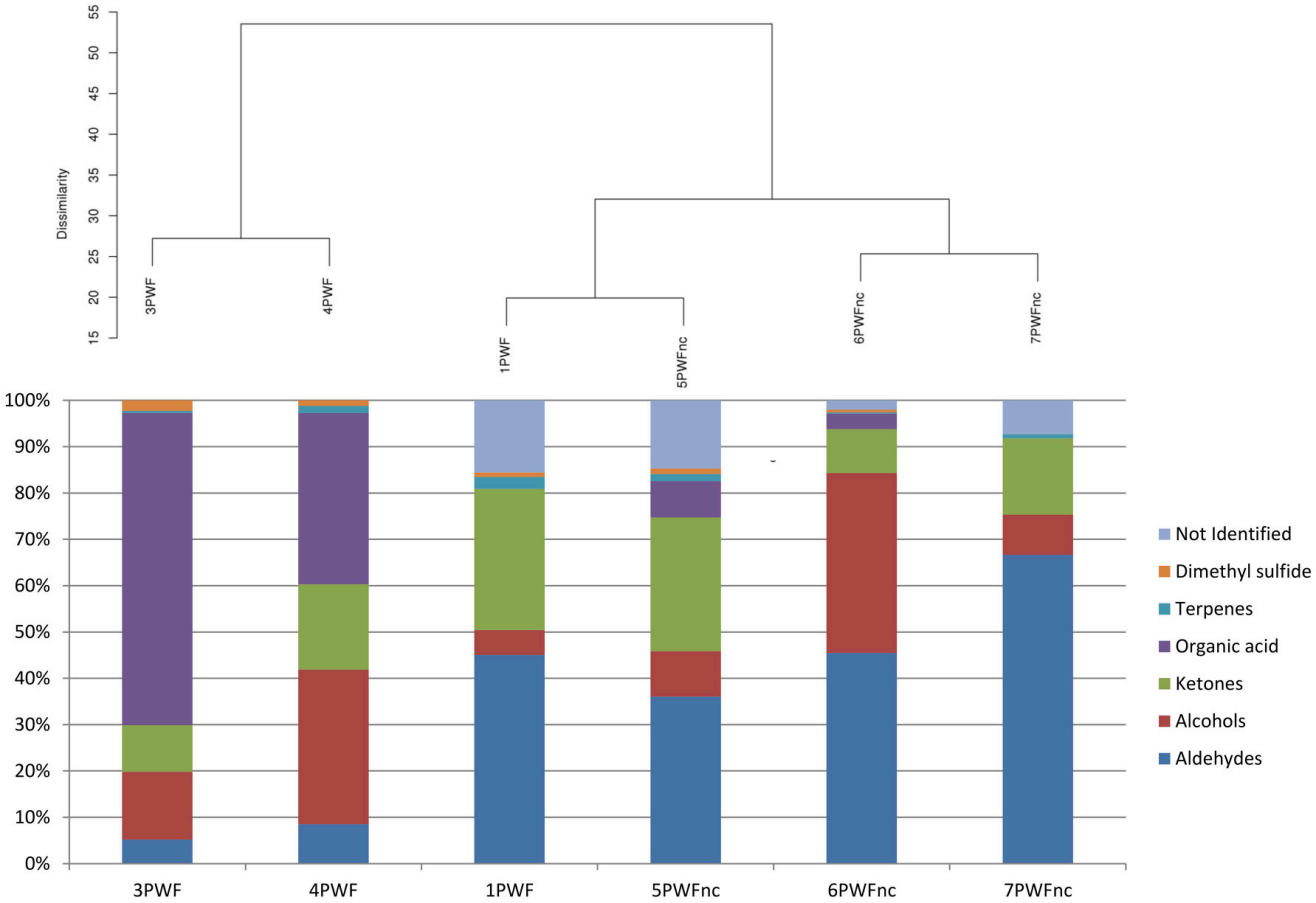
**Hierarchical clustering analysis of whey samples based on the headspace compound composition**. The percentage of each group of volatile compounds are presented in the bars.

The headspace of six whey samples (1PWF, 3PWF, 4PWF, 5 PWFnc, 6PWFnc, and 7 PWFnc as reported in Table 1) were analyzed using SPME-GC/MS analysis. The compounds measured in the whey samples were grouped based on their chemical natures (listed in detail in Table S2). In Figure 3, the whey samples are grouped based on the statistical clustering of the headspace compound composition that is represented in the bars. The samples are divided into two clusters; the first contained 3PWF and 4PWF, and the second contained all of the PWFnc samples and the 1PWF sample. In this last cluster, aldehydes, alcohols and ketones (the products of lipid oxidation) were predominant, whereas these compounds were present in the 3PWF and 4PWF samples in lower concentrations. The 3PWF and 4PWF samples contained a relevant amount of organic acids (67.4 and 37.0%, respectively) that was reduced or absent in all of the PWFnc and 1PWF samples. The presence of two characteristic fermentation products [2,3-butanedione (diacetyl) and 3-hydrozy-2-butanone (acetoin)] was detected in 3PWF, 4PWF, 5PWFnc, and 6PWFnc.

### Correlation between the microbial community and volatile compounds in whey samples

**Figure 4:**
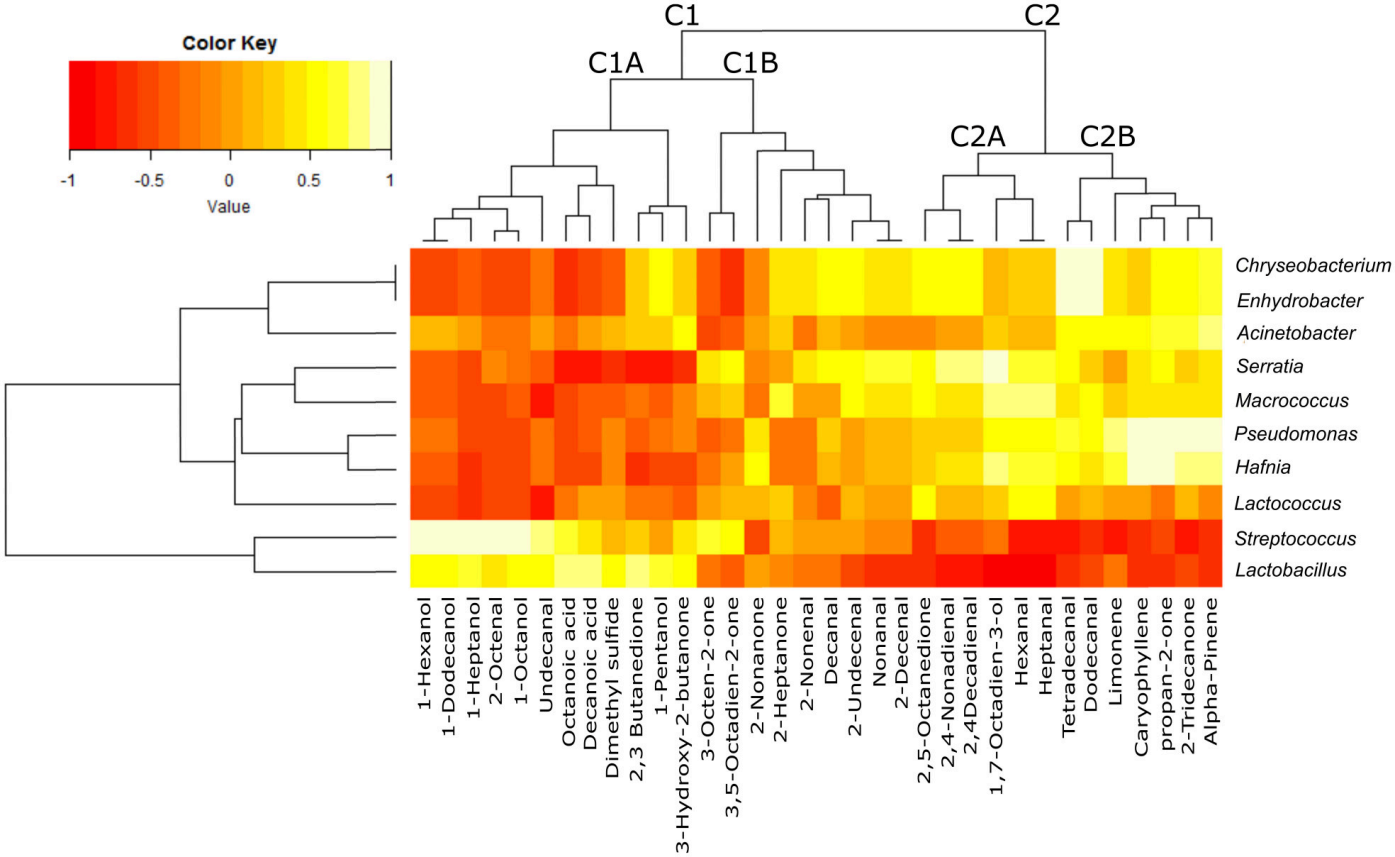
**Heatmap plot reporting the Spearman's *rho* statistical correlation between the abundance of bacterial genera and the abundance of volatile compounds in the whey samples**. The color key is reported on the top, with −1 (dark red) indicating negative correlation and +1 (light yellow) indicating positive correlation.

The heatmap obtained merging microbial community and chemical compound abundance (Figure 4) showed that the volatile compounds clustered into two groups (C1 and C2), each of which was separated into two sub-clusters (C1A, C1B, C2A, and C2B). Most of the compounds contained in cluster C1A (which contained long chain primary alcohols, organic acids, methyl ketones, dimethylsulfide, acetoin and diacetyl) were strongly positively correlated with the genera *Lactobacillus* and *Streptococcus*. These two lactic acid bacteria used as the starters were predominant in the community of whey samples, but the correlation appeared to be negative or was not relevant with the third starter genus *Lactococcus* or the spoiler bacteria (*Pseudomonas, Serratia, Acinetobacter, Enhydrobacter*, and *Chryseobacterium*). In contrast, cluster C2, which included the products of lipid peroxidation such as aldehydes, secondary alcohols, methyl ketones (C2A) and terpenes (C2B), was negatively correlated with the *Lactobacillus* and *Streptococcus* genera but strongly positively correlated with the spoiler bacteria.

## Discussion

The sampling of 1PW–4PW lots and the analysis using culture-dependent methods were conducted to establish a background of the microbiological quality of these whey samples before proceeding with the community characterization using the NGS methodology.

The bacterial counts for 1PW–4PW lots confirmed the reports by the dairy factory. The total mesophilic bacteria count (TMC) was very high for all samples, with values of approximately 10^6^–10^7^ CFU/mL. These results are very similar to the bacterial load of pasteurized whey reported previously (Lo et al., 2016).

The high concentration of bacteria could partially derive from the bacterial load present in milk at the time of its use for dairy production but could also be due to contamination downstream in the dairy chain. Several studies conducted on milk showed mesophilic bacteria counts ranging between 10^2^ and 10^7^ CFU/mL with a population that was primarily composed of lactic acid bacteria, including the *Lactococcus* and *Lactobacillus* genera, as well as *Enterobacteriaceae* and *Micrococcaceae*, which are partially resistant to thermal treatments (Quigley et al., 2012). The number of aerobic spores was very high, with counts of 10^3^–10^4^ CFU/mL. Aerobic spores produced primarily by *Bacillaceae* and *Paenibacillaceae* are commonly found in silage and can withstand heat treatment and persist in raw dairy materials (Ivy et al., 2012). Contamination with these spores can lead to bacterial growth and the premature deterioration of whey and products derived from whey, such as Ricotta, as demonstrated by the analysis of the Ricotta produced using the PW lots. In these Ricotta samples, the predominant community was composed of the *Bacillus* and *Paenibacillus* genera (Sattin et al., 2016). Enzymes produced by *Bacillus* spp., such as protease, lipase and phospholipase, can cause changes in the consistency of the products and typical alterations in the aroma and flavor (Heyndrickx et al., 2010).

The introduction of a specific medium for *Pseudomonadaceae* to the analysis of the last Lots (5PW–8PW) revealed a high number of bacteria belonging to this family, with a bacterial load between 10^4^ and 10^7^ CFU/mL. Microorganisms belonging to the *Pseudomonas* genus may be responsible for the release of heat-resistant proteolytic enzymes involved in the degradation of the finished product or the raw materials themselves (Marchand et al., 2009; De Jonghe et al., 2011). Additionally, *Bacillus* and *Pseudomonas* could be involved in the production of pigments visible in the finished product (Martin et al., 2011; Andreani et al., 2013; Sattin et al., 2016). Indeed, Ricotta discoloration defects were reported by the dairy factory (Sattin et al., 2016).

NGS technology is a powerful tool to study the microbial communities of foods in depth and is considered a complementary technique to microbiological culture-dependent methods. High-throughput 16S rRNA gene library sequencing robustly determines the diversity and abundance of microbial communities in a quantitative and qualitative manner. Therefore, the integration of the microbiological data with the NGS analysis might provide a global overview of the microbial communities. For this reason, NGS analysis was applied to evaluate the composition of the microbial communities in the whey lots sampled for the microbiological analysis. RNA was the preferred template for the analysis of the living microbiota. The same approach was also used to analyze the microbial communities in whey lots collected and frozen in the factory during the summer of 2013 but DNA was inevitably used as the template for library construction for the NGS analysis. Part of these frozen whey samples were defective for organoleptic properties and were excluded from production.

The results demonstrate high biodiversity in the whey microbiota that was particularly evident in the community obtained starting from the RNA template. In all samples (from both the RNA and DNA templates), the community was composed of the starter lactic acid bacteria (derived from milk and partially resistant to pasteurization, such as *Lactococcus, Streptococcus*, and *Lactobacillus*), contaminating bacteria (*Serratia, Pseudomonas*, and *Acinetobacter*) and spore-forming bacteria (*Bacillus*). The results obtained with the NGS analysis mostly agreed with those obtained by the Sanger sequencing of isolated colonies.

*Gammaproteobacteria*, especially *Pseudomonadaceae, Shewanellaceae, Moraxellaceae*, and *Enterobacteriaceae*, were present in all of the communities but in variable amounts. These bacteria are typical environmental contaminants that are usually completely destroyed by the correct pasteurization process; therefore, secondary contamination during the pasteurized whey shelf-life may be responsible. An alternative explanation is that contamination occurred in whey collected after cheesemaking. These contaminations can produce very high microbiological loads (more than 10^7^ cell/mL) that are not completely eliminated by pasteurization. Therefore, the bacteria surviving the heat treatment can grow in pasteurized whey if it is not adequately stored and managed. The current regulations and guidelines for the storage and management of whey that consider it a by-product are not very effective. Thus, contaminating bacteria and spoilage are frequently reported.

In the NGS data, spore-forming microorganisms were present in 0.03–20% of the PW samples. Sample 8PW, which contained 20% *Anoxybacillus*, had the highest aerobic spore counts (one or two logs more than the other PW samples). The spore-forming community of the PWF samples was between 0 and 0.05%; however, this community is probably composed by the vegetative forms being the extraction of RNA from spores a challenging point. In fact the presence of active metabolism with RNA biosynthesis in the spores is uncertain and controversial. Additionally, the extraction of nucleic acids from spores is challenging because spores are rarely lysed by the commonly used lysis buffers in DNA/RNA extraction kits (Thomas et al., 2013; Mertens et al., 2014). Therefore, the spore counts deduced by the NGS analysis might not be accurate.

The statistical analysis of β-diversity considering all NGS-analyzed whey samples demonstrated that the communities derived from the RNA and DNA templates were different despite the small number of samples tested (see Figure 2). Because the whey was pasteurized, this finding could be of great importance for the living microbiota. DNA fragments from the bacteria that died during pasteurization might still remain and be amplified. For these reasons, the use of an RNA template is absolutely preferred when available, especially for technologically treated or fermented foods (Carraro et al., 2011; De Pasquale et al., 2014).

Unfortunately, although DNA should be used to analyze the defective whey samples, the statistical analysis demonstrated that the DNA communities lacked (completely or in part) 38 OTUs, most of which were identified as contaminant bacteria (*Gammaproteobacteria*). The major biodiversity of the living microflora pictured using RNA as template based on the diversity indices (see Table 2) might be representative of the major biodiversity in the living contaminant bacteria. However, we could not exclude the possibility that the different extraction methods (DNA or RNA) or freezing of the samples prior to extraction modified the nucleic acid yield for some taxa (Rubin et al., 2013; Larsen et al., 2015).

A significant difference between normal and defective whey communities was obtained when all samples or only the DNA samples were considered as reported in Table 3. In this last comparison in which the possible confounding effects were reduced, most *Gammaproteobacteria* OTUs differentiated the communities. These data indicate that most *Gammaproteobacteria* are environmental contaminant bacteria that may represent the real variability acting on the spoilage of whey.

The GC/MS analysis demonstrated different profiles between normal and defective whey (Figure 3) despite one normal sample (1PWF) clustering with the defective samples. The PCoA analysis based on microbial community profiles clustered 1PWF with 3PWF and 4PWF (Figure 2). The microbial community composition of 1PWF was composed in large part of *Pseudomonadales* (*Gammaproteobacteria*), whereas the PWFnc (excluding 9PWFnc, which was not included in the GC analysis) contained less *Pseumononadales* and more lactic acid bacteria. Sample 1PWF was identified as normal by the factory and was used to produce a normal Ricotta despite a result from the volatile compound composition that classified it as defective. However, the particular microbial population suggested that some specific compounds derived by the *Pseudomonadales* community could have modified the perception of the organoleptic properties (this sample contained aldehydes similar to the PWFnc samples but more ketones and more than 15% unidentified compounds). Aldehydes, alcohols and ketones, which are principally produced via lipid oxidation, are predominant in all PWFnc samples (and 1PWF). These compounds are present in the PWF samples in lesser amounts, and their presence may be a consequence of whey pasteurization or bacterial metabolism. Indeed, the accumulation of free fatty acids is the result of the microbial lipase and esterase actions on the endogenous lipolytic enzymes of whey. *Lactobacillus, Streptococcus*, and *Pseudomonas* strains might have an intense lipolytic action on triglycerides (Remenant et al., 2015). Moreover, the oxidizing environment where aldehydes are formed is strictly correlated with the metabolic needs of the spoiler bacteria found in the present study, which are aerobic or facultative anaerobic. The lipid oxidation products are primarily comprised in cluster C2 and positively correlated with the spoiler bacteria (Figure 4).

Hexanal, which is typical marker of oxidative processes, has an intrinsic tallowy and leafy green smell with a threshold of perception that is generally 10 times higher than 2,4-decadienal, which has an intrinsic frying odor. The series of methyl ketones with between 5 and 13 carbon atoms have a flavor that resembles that of fruit, whereas propan-2-one has an acetone flavor (Belitz and Grosch, 1999).

2,3-butanedione (diacetyl) and 3-hydroxy-2-butanone (acetoin) are present in variable amounts or are absent in whey samples. Diacetyl is obtained from the condensation of two molecules of pyruvate during the valine and leucine biosynthesis pathways (Belitz and Grosch, 1999). In a reducing environment, diacetyl is reduced to acetoin. Both compounds are key odors of the butter. Their production is primarily due to the activity of lactic acid bacteria, especially *Lactococcus lactis* ssp. *lactis biovar diacetylactis* (Mauriello et al., 2001). The common substrate for diacetyl synthesis is citrate. In the manufacture of mozzarella cheese, citric acid is usually employed for the clotting of casein, and a variable quantity of citrate remains in the serum (Gallardo-Escamilla et al., 2005; Di Cagno et al., 2014). The amount of these volatile compounds in whey samples depends on the prevalence of whey derived by mozzarella production; however, the genus *Lactococcus* does not seem to correlate with these compounds (Figure 4). This result could be explained by the finding that all species and strains belonging to the *Lactococcus* genus are included in these OTUs. The ability to discriminate different species/strains belonging to the same genus is the limit of the currently available NGS methodology because the genus level is the deepest level with an acceptable classification performance for short sequencing reads (Mizrahi-Man et al., 2013). Volatile compound production is often a strain-specific trait; therefore, a more in depth identification of bacterial strains is essential to obtain a more accurate result. The *Lactococcus* shown in Figure 4 appears to be similar to the spoiler bacteria possibly due to the spoilage activity of several *Lactococcus* species and strains (Ledenbach and Marshall, 2009; Remenant et al., 2015) but may be more simply due to the inverse correlation in abundance between the two most common starter genera (*Lactococcus and Streptococcus* as clearly shown in Figure 1).

*Chryseobacterium* and *Enhydrobacter* correlate with volatile compounds in cluster C2 and result in overrepresentation of the PWFs_nc in Table 5. The bacteria were previously reported as spoilage bacteria involved in lipid oxidation and off-odor production (Callewaert et al., 2014; Bekker et al., 2016). *Chyseobacterium* was frequently isolated from the ultrafiltration and reverse osmosis membranes used in dairy plants (Tang et al., 2009; Marino et al., 2013).

In conclusion, the integrated analysis of the microbiota composition and volatile compounds demonstrated a very poor quality of whey. Heat treatment (i.e., pasteurization) is essential to extend the shelf-life of the whey but may be partially responsible for lipid peroxidation; therefore, it should be used with care. Different treatments, such as bactofugation or microfiltration, might be better solutions to improve the quality of the whey and increase its value. However, the microbiological quality of the whey before treatments should be improved to avoid initiating the spoilage process. The current regulations and guidelines for the storage and management of whey need to be revised in consideration of both its actual high commercial value and to improve food safety.

The results reported in this study demonstrate the possibility of integrating different –omics data to provide the most complete view of the sample composition. Knowing the composition of the food microbiome is very important to define the safety and quality of a food product. NGS platforms are an interesting approach for food microbiology because they allow deep microbial community definitions based directly on food samples (Ercolini, 2013; Mayo et al., 2014). The strong competition between manufacturers has resulted in sustained technical improvements and cost reduction of almost all NGS platforms, thereby allowing wider usage of these technologies. The correlation of the volatile compound composition with the microbial community opens interesting opportunities to dissect the spoilage events that occur in food products and to identify the bacteria involved.

The results obtained in the present study demonstrate how the application of integrated -omics technologies can become a suitable tool in the food industry in the future for the improvement of the quality and safety of products.

## Author contributions

ES, NA, LC, SB made all the experimental activities. LF and RL were involved in sampling. AT, EN, BS were involved in data analysis. ES, LC, and BC writing of the manuscript. BC supervision of all the study.

### Conflict of interest statement

The authors declare that the research was conducted in the absence of any commercial or financial relationships that could be construed as a potential conflict of interest.
