# Supplementary material for: A Multi-Omics Approach to Evaluate the Quality of Milk Whey Used in Ricotta Cheese Production
Source: Front Microbiol. 2016 Aug 17;7:1272. doi: 10.3389/fmicb.2016.01272 (PMC4987355; doi:10.3389/fmicb.2016.01272)
Supplement: Supplementary file 1 [file Table1.docx]

|  | **Genus or species (16S sequencing)** | **Isolation medium** |
| --- | --- | --- |
| **1** | *Hafnia alvei* | Milk PCA |
| **2** | *Lactococcus lactis subsp. cremoris* | Milk PCA |
| **3** | *Hafnia alvei* | Milk PCA |
| **4** | *Microbacterium sp.* | Milk PCA |
| **5** | *Bacillus sp.* | Milk PCA |
| **6** | *Streptococcus thermophilus* | Milk PCA |
|  |  |  |
| **7** | *Streptococcus thermophilus* | Milk PCA |
| **8** | *Lactococcus lactis* | Milk PCA |
| **9** | *Hafnia alvei* | Milk PCA |
| **10** | *Hafnia alvei* | Milk PCA |
| **11** | *Lactococcus lactis* | Milk PCA |
| **12** | *Bacillus licheniformis* | Milk PCA |
| **13** | *Streptococcus* | Milk PCA |
| **14** | *Streptococcus macedonicus* | Milk PCA |
| **15** | *Shewanella* | Milk PCA |
| **16** | *Kocuria rhizophila* | MYP Pink-yellow |
| **17** | *Macrococcus caseolyticus* | MYP Light pink |
| **18** | *Macrococcus caseolyticus* | MYP Light pink |
| **19** | *Lactococcus lactis* | MYP Transparent |
| **20** | *Kocuria rhizophila* | MYP Pink-orange |
| **21** | *Lactococcus lactis* | MYP Transparent |
| **22** | *Lactococcus lactis* | MYP Transparent |
| **23** | *Bacillus cereus* | MYP Light pink |
| **24** | *Bacillus cereus* | Milk PCA+starch |
| **25** | *Uncultured Streptococcus* | Milk PCA+starch |
| **26** | *Bacillus cereus* | Milk PCA+starch |
| **27** | *Bacillus cereus* | Milk PCA+starch |
| **28** | *Leuconostoc mesenteroides* | Milk PCA+starch |
| **29** | *Bacillus licheniformis* | Milk PCA+starch |
|  |  |  |

**Table S1**. Species identification by BLAST comparison ( > 98%) of 16S gene sequences of isolate colonies from 3PW and 4PW samples of Whey. For MYP isolates the color of the colonies is reported.
