# Supplementary material for: A Multi-Omics Approach to Evaluate the Quality of Milk Whey Used in Ricotta Cheese Production
Source: Front Microbiol. 2016 Aug 17;7:1272. doi: 10.3389/fmicb.2016.01272 (PMC4987355; doi:10.3389/fmicb.2016.01272)
Supplement: Supplementary file 2 [file Table2.DOCX]

|  | **3PWF** | **4PWF** | **1PWF** | **5PWFnc** | **6PWFnc** | **7PWFnc** |
| --- | --- | --- | --- | --- | --- | --- |
| ***Aldehydes*** |  |  |  |  |  |  |
| Hexanal | 0.11 | 0.50 | 5.47 | 1.27 | 0.10 | 10.73 |
| Heptanal | 0.11 | 0.14 | 2.39 | 0.25 | 0.00 | 9.79 |
| 2-Octenal | 1.96 | 2.78 | 0.00 | 0.00 | 6.46 | 1.06 |
| Nonanal | 0.19 | 0.45 | 4.24 | 0.99 | 8.91 | 8.93 |
| 2-Nonenal | 0.24 | 0.04 | 2.95 | 0.37 | 14.42 | 9.79 |
| Decanal | 0.04 | 0.13 | 0.92 | 0.28 | 1.82 | 0.40 |
| 2,4-Nonadienal | 0.01 | 0.05 | 0.58 | 0.10 | 0.51 | 2.01 |
| 2-Decenal | 0.30 | 0.43 | 2.21 | 1.36 | 3.27 | 4.05 |
| Undecanal | 0.64 | 1.16 | 0.53 | 0.41 | 2.12 | 0.37 |
| Dodecanal | 0.85 | 1.93 | 11.47 | 19.40 | 3.52 | 10.54 |
| 2,4-Decadienal | 0.03 | 0.14 | 2.28 | 0.56 | 1.27 | 3.05 |
| 2-Undecenal | 0.40 | 0.44 | 1.89 | 1.96 | 2.60 | 4.68 |
| Tetradecanal | 0.29 | 0.35 | 10.09 | 9.11 | 0.47 | 1.25 |
| ***Total*** | ***5.16*** | ***8.52*** | ***45.03*** | ***36.05*** | ***45.48*** | ***66.65*** |
| ***Alcohols*** |  |  |  |  |  |  |
| 1-Pentanol | 0.32 | 0.22 | 0.26 | 0.45 | 0.43 | 0.10 |
| 1-Hexanol | 6.56 | 16.08 | 2.24 | 4.88 | 15.34 | 2.86 |
| 1-Heptanol | 3.36 | 5.17 | 0.69 | 1.76 | 10.85 | 1.70 |
| 1,7-Octadien-3-ol | 1.04 | 2.09 | 1.81 | 1.29 | 1.17 | 2.11 |
| 1-Octanol | 2.21 | 3.52 | 0.37 | 0.70 | 7.61 | 1.72 |
| 1-Dodecanol | 1.15 | 6.30 | 0.00 | 0.69 | 3.44 | 0.16 |
| ***Total*** | ***14.65*** | ***33.38*** | ***5.36*** | ***9.77*** | ***38.85*** | ***8.65*** |
| ***Ketones*** |  |  |  |  |  |  |
| Propan-2-one | 4.37 | 12.87 | 17.61 | 7.38 | 4.52 | 5.49 |
| 2,3-butanedione | 0.71 | 0.36 | 0.00 | 3.04 | 2.02 | 0.00 |
| 3-hydroxy-2-butanone | 0.01 | 0.04 | 0.00 | 10.38 | 1.05 | 0.00 |
| 2-Heptanone | 0.05 | 0.12 | 0.00 | 0.67 | 0.25 | 0.57 |
| 2,5-Octanedione | 0.26 | 0.22 | 2.67 | 1.20 | 0.36 | 3.65 |
| 3-Octen-2-one | 0.02 | 0.05 | 0.00 | 0.00 | 0.14 | 1.28 |
| 2-Nonanone | 3.82 | 1.85 | 5.52 | 1.82 | 0.39 | 1.27 |
| 3,5-octadien-2-one | 0.23 | 0.47 | 0.10 | 0.00 | 0.40 | 1.93 |
| 2-Tridecanone | 0.58 | 2.42 | 4.60 | 4.41 | 0.36 | 2.31 |
| ***Total*** | ***10.05*** | ***18.41*** | ***30.50*** | ***28.89*** | ***9.50*** | ***16.50*** |
| ***Organic acid*** |  |  |  |  |  |  |
| Octanoic acid | 30.07 | 20.76 | 0.00 | 1.63 | 1.74 | 0.00 |
| Decanoic acid | 37.35 | 16.21 | 0.00 | 6.22 | 1.54 | 0.00 |
| ***Total*** | ***67.42*** | ***36.96*** | ***0.00*** | ***7.85*** | ***3.28*** | ***0.00*** |
| ***Terpenes*** |  |  |  |  |  |  |
| Alpha-pinene | 0.07 | 0.57 | 0.76 | 0.62 | 0.09 | 0.38 |
| Limonene | 0.19 | 0.51 | 0.99 | 0.60 | 0.08 | 0.18 |
| Caryophyllene | 0.15 | 0.45 | 0.85 | 0.33 | 0.12 | 0.29 |
| ***Total*** | ***0.42*** | ***1.54*** | ***2.59*** | ***1.54*** | ***0.29*** | ***0.85*** |
| ***Aliphatic sulfur compounds*** |  |  |  |  |  |  |
| Dimethyl sulfide | 2.30 | 1.19 | 0.92 | 1.16 | 0.60 | 0.00 |
|  |  |  |  |  |  |  |
| Not Identified | 0.00 | 0.00 | 15.60 | 14.74 | 2.01 | 7.34 |

**Table S2.** Head space volatile compounds detected in PWF and PWFnc samples with GC/MS (all the values are reported as %).
